# Mid-Term Results of the Multicenter CAMPARI Registry Using the E-Liac Iliac Branch Device for Aorto-Iliac Aneurysms

**DOI:** 10.3390/jcdd13010048

**Published:** 2026-01-15

**Authors:** Francesca Noce, Giulio Accarino, Domenico Angiletta, Luca del Guercio, Sergio Zacà, Mafalda Massara, Pietro Volpe, Antonio Peluso, Loris Flora, Raffaele Serra, Umberto Marcello Bracale

**Affiliations:** 1 Vascular Surgery Unit, Department of Public Health, Federico II University Hospital, 80138 Naples, Italy; f.noce@studenti.unina.it (F.N.); giuaccarino@unisa.it (G.A.); luca.delguercio@unina.it (L.d.G.); umbertomarcello.bracale@unina.it (U.M.B.); 2 Department of Emergency and Organ Transplants, Giovanni XXIII University Hospital, 70121 Bari, Italy; domenico.angiletta@uniba.it (D.A.); sergiozac89@gmail.com (S.Z.); 3 Divisions of Vascular Surgery, Bianchi-Melacrino Metropolitan Hospital, 89124 Reggio Calabria, Italy; mafalda.massara@ospedalerc.it (M.M.); pietrovolpe257@gmail.com (P.V.); 4 Vascular Surgery, Hospital of National Importance San Giuseppe Moscati, 83100 Avellino, Italy; antonio.peluso@aornmoscati.it (A.P.); loris.flora@aornmoscati.it (L.F.); 5 Interuniversity Center of Phlebolymphology (CIFL), International Research and Educational Program in Clinical and Experimental Biotechnology, University “Magna Graecia” of Catanzaro, 88100 Catanzaro, Italy; 6 Department of Medical and Surgical Sciences, University “Magna Graecia” of Catanzaro, 88100 Catanzaro, Italy

**Keywords:** aorto-iliac aneurysm, iliac branch device, EVAR, CAMPARI study

## Abstract

**Background**: Intentional occlusion of the internal iliac artery (IIA) during endovascular repair of aorto-iliac aneurysms may predispose patients to pelvic ischemic complications such as gluteal claudication, erectile dysfunction, and bowel ischemia. Iliac branch devices (IBDs) have been developed to preserve hypogastric perfusion. E-Liac (Artivion/Jotec) is one of the latest modular IBDs yet reports on mid-term performance are limited to small single-center cohorts with short follow-up. The CAMpania PugliA bRanch IliaC (CAMPARI) study is a multicenter investigation of E-Liac outcomes. **Methods**: A retrospective observational cohort study was conducted across five Italian vascular centers. All consecutive patients undergoing E-Liac implantation for aorto-iliac or isolated iliac aneurysms between January 2015 and December 2024 were identified from prospectively maintained registries. Inclusion criteria comprised elective or urgent endovascular repair of aorto-iliac aneurysms in which an adequate distal sealing zone was not available without covering the IIA and suitability for the E-Liac device according to its instructions for use (IFU). Patients with a life expectancy < 1 year or hostile anatomy incompatible with the IFU were excluded. The primary end point was freedom from branch instability (occlusion/stenosis, kinking, or detachment of the bridging stent). Secondary end points included freedom from any endoleak, freedom from device-related reintervention, freedom from gluteal claudication, aneurysm-related and all-cause mortality, acute renal failure, and sac regression > 5 mm. **Results**: A total of 69 consecutive patients (68 male, 1 female, median age 72.0 years) received 74 E-Liac devices, including 5 bilateral implantations. The mean infrarenal aortic diameter was 45 mm and the mean CIA diameter 34 mm; 14 patients (20.0%) had a concomitant IIA aneurysm (>20 mm). Concomitant fenestrated or branched aortic repair was performed in 23% of procedures. Two patients received a standalone IBD without implantation of a proximal aortic endograft. Technical success was achieved in 71/74 cases (96.0%); three failures occurred due to inability to catheterize the IIA. Distal landing was in the main IIA trunk in 58 cases and in the posterior branch in 13 cases. Over a median follow-up of 18 (6; 36) months, there were four branch instability events (5.4%): three occlusions and one bridging stent detachment. Seven patients (9.5%) developed endoleaks (one type Ib, two type II, two type IIIa, and two type IIIc). Five patients (6.8%) required reintervention, and five (6.8%) reported gluteal claudication. There were seven all-cause deaths (10%), none within 30 days or related to aneurysm rupture; causes included COVID-19 pneumonia, acute coronary syndrome, melanoma, gastric cancer, and stroke. No acute renal or respiratory failure occurred. Kaplan–Meier analysis showed 92% (95% CI 77–100) freedom from branch instability in the main-trunk group and 89% (60–100) in the posterior-branch group (log-rank *p* = 0.69). Freedom from any endoleak at 48 months was 87% (95% CI 75–95), and freedom from reintervention was 93% (95% CI 83–98). **Conclusions**: In this multicenter cohort, the E-Liac branched endograft demonstrated high technical success and favorable early–mid-term outcomes. Preservation of hypogastric perfusion using E-Liac was associated with low rates of branch instability, endoleak, and reintervention, with no 30-day mortality or aneurysm-related deaths. These findings support the safety and efficacy of E-Liac for aorto-iliac aneurysm management, although larger prospective studies with longer follow-up are needed.

## 1. Introduction

Endovascular aneurysm repair (EVAR) has become the preferred modality for treating aorto-iliac aneurysms, offering reduced peri-operative morbidity and mortality compared with open repair [1,2,3]. Nonetheless, extension of aneurysmal disease into the iliac system is common, occurring in up to 40% of infrarenal aneurysms [4,5]. When a suitable sealing zone is only available in the external iliac artery (EIA), traditional EVAR techniques achieve distal sealing by intentional coverage or embolization of the internal iliac artery (IIA) [6], exposing patients to pelvic ischemia manifesting as buttock claudication, erectile dysfunction, or colonic ischemia. Reported rates of buttock claudication following hypogastric occlusion range from 30% to 55% [7]. Importantly, the incidence and severity of buttock claudication after internal iliac artery occlusion are strongly influenced by whether occlusion is unilateral or bilateral, with significantly higher rates reported after bilateral hypogastric exclusion [8]. To preserve hypogastric flow, modular iliac branch devices (IBDs) have been developed. They were first employed in clinical practice in 2006 [9], and they offer the advantage of achieving an adequate distal sealing zone during EVAR without the need for intentional occlusion of the IIA. Early experiences with the Gore Excluder IBE and Cook Zenith IBD demonstrated favorable technical success and patency rates but were limited by rigid delivery systems and strict anatomic requirements. To date, several iliac branch devices have been developed over the past two decades. Early-generation platforms such as the Zenith Branch Iliac (ZBIS) and the Gore Excluder IBE demonstrated high technical success and durable preservation of hypogastric perfusion, albeit with stricter anatomic requirements and more challenging navigation in tortuous iliac segments [10]. Large multicenter series and systematic reviews have reported primary patency rates consistently above 90% and low incidences of pelvic ischemic complications for these devices. More recent comparative analyses suggest that mid-term outcomes across different IBD platforms are broadly comparable, with device selection largely driven by center experience and patient-specific anatomy. Within this landscape, the E-Liac (Artivion/Jotec), introduced in 2014, represents a newer-generation, more flexible alternative designed to expand anatomic applicability and facilitate branch cannulation. While E-Liac has been described as a more trackable and adaptable platform, our retrospective registry does not include a structured assessment of anatomic suitability for alternative IBDs; therefore, we cannot quantify how many patients would have specifically benefited from device flexibility. This remains a relevant topic for future prospective comparative registries. Moreover, the E-Liac is the only device currently available with a specific indication for standalone treatment of CIA aneurysms [11]. Early series reported encouraging results but were limited by small sample sizes and a follow-up of typically ≤12 months [4,12]. Larger mid-term and long-term series have recently become available, including a single-center mid-term E-Liac experience [13] and an 11-year long-term cohort, [14] as well as comparative analyses versus the Cook ZBIS device [4,10]. To address this knowledge gap, the CAMpania PugliA bRanch Iliaco (CAMPARI) (ClinicalTrials.gov ID: NCT06946381) study collects mid-term outcomes from a consecutive cohort of patients treated with the E-Liac device in several high-volume centers.

## 2. Materials and Methods

This is a retrospective, multicenter study including consecutive patients treated between January 2015 and December 2024 at five Vascular Surgical centers. The present study was conducted in accordance with the Declaration of Helsinki and approved by the Institutional Review Board of the Interuniversity Centre of Phlebolymphology (CIFL ER.ALL.2014.11.A). The study is registered at ClinicalTrials.gov (NCT06946381). All consecutive patients undergoing E-Liac implantation for aorto-iliac or isolated iliac aneurysms were identified from prospectively maintained registries. The cohort includes unilateral and bilateral procedures. Baseline demographics, comorbidities, anatomic measurements, procedural details, and outcomes were recorded in a dedicated database.

Eligible patients had an indication for endovascular repair of an aorto-iliac or isolated iliac aneurysm and met the E-Liac IFU: infrarenal neck suitable for standard EVAR, CIA diameter between 20 and 40 mm with length ≥ 20 mm, and IIA diameter ≤ 12 mm and length ≥ 20 mm without significant calcification or thrombus. Aneurysm extension into the CIA requiring hypogastric preservation was mandatory; patients with abdominal aortic aneurysm not involving the iliac arteries were excluded. Aneurysms of the IIA were defined as a maximum diameter > 20 mm. Patients with a life expectancy < 1 year or hostile iliac anatomy (excessive angulation, severe calcification) were excluded, as well as patients refusing to be enrolled in the registry.

Pre-operative planning was based on standard arterial-phase computed tomography angiography, with thin-slice reconstructions (≤1 mm) routinely used for three-dimensional workstation analysis and centerline measurements. All procedures were performed by experienced vascular surgeons under general, spinal, or local anesthesia. Standard modular EVAR was undertaken using a bifurcated main body in cases of aorto-iliac aneurysms; two patients with isolated CIA aneurysms received the IBD alone. All procedures were planned with the intent to preserve at least one internal iliac artery, and no cases of intentional bilateral hypogastric occlusion were performed during the index procedure.

Completion angiography using digital subtraction angiography (DSA) assessed patency and endoleaks. Technical success was defined as deployment of the IBD with preservation of IIA flow and absence of endoleak on completion angiography. When a second bridging stent was used, its model and dimensions were recorded.

Patients underwent CTA at 1-month and at 6-month intervals during the first year and then annually. Imaging studies were reviewed to assess endograft patency, endoleaks, branch instability, and sac diameter. CTA data were centralized to the leading center (Federico II University Hospital) for independent evaluation of the study outcomes.

The primary end point was branch instability, defined as occlusion, severe stenosis (>50%), or kinking or detachment of the iliac branch or bridging stent. Secondary end points included any endoleak (classified as type I, II, or III according to their source), reintervention related to the device or aneurysm, gluteal claudication, sac regression > 5 mm, acute kidney injury (as defined according to the KDIGO criteria, incorporating both serum creatinine changes and estimated glomerular filtration rate (eGFR)), and acute respiratory failure requiring mechanical ventilation. Deaths were categorized as aneurysm-related or unrelated; 30-day/in-hospital mortality was also recorded.

Continuous variables were checked for normality using the Shapiro–Wilk test. Normally distributed variables are reported as mean ± standard deviation, whereas non-normal variables are reported as median [interquartile range (IQR)]. Categorical variables are presented as counts and percentages. Comparisons between groups were made using the χ^2^ or Fisher’s exact test for categorical variables and the Mann–Whitney U test for continuous variables. Kaplan–Meier survival analysis was used to estimate freedom from branch instability, any endoleak, and reintervention. Curves were truncated when fewer than 10 subjects remained at risk to avoid unstable estimates. Differences were assessed using the log-rank test. A two-sided *p*-value < 0.05 was considered statistically significant. Statistical analyses were performed using JASP software version 0.18.3 (JASP Team, Amsterdam, The Netherlands). Follow-up data were complete for all primary and secondary outcomes. Occasional missing values were observed for selected peri-operative variables and were handled using available-case analysis. Percentages are reported using the appropriate denominator: patient-level variables are expressed as proportions of the total number of patients (*n* = 69), whereas device-level variables are expressed as proportions of the total number of implanted devices (*n* = 74).

The study was carried out in accordance with the Strengthening the Reporting of Observational Studies in Epidemiology (STROBE) guidelines for observational research [15].

## 3. Results

A total of 69 patients (68 male) underwent implantation of 74 E-Liac devices.

In 48 (64.8%) cases, the aortic diameter was ≤50 mm; therefore, the implantation of an IBD combined with an EVAR was guided by insufficient iliac sealing zone. Baseline characteristics are summarized in Table 1.

The median age was 72 (67–78) years. Cardiovascular risk factors were common: 89.9% were hypertensive, 71% were dyslipidemic, and 36.2% had chronic obstructive pulmonary disease. Fourteen patients (20.3%) had an IIA aneurysm (>20 mm). The median body mass index was 26.0 (25.0–27.7) kg/m^2^.

Anatomic measurements are shown in Table 2.

The median infrarenal aortic diameter was 44 (33–55) mm and the CIA diameter 34 (30–41) mm; the median CIA length was 57 (51–68) mm. The median IIA diameter was 10 (8–12) mm and the length 32 (22–40) mm. Fourteen patients (20.0%) had a diagnosed IIA aneurysm (>20 mm).

A total of 86 bridging stent grafts were implanted; most devices required a single bridging stent, while a second stent was used in selected cases, detailed in Table 3.

The identical median stent length observed reflects the use of standardized commercially available bridging stent graft lengths, with 57 mm representing the most frequently selected configuration in routine clinical practice. The primary bridging stent was the E-Ventus in 66.3% of cases, BeGraft (Bentley InnoMed GmbH, Hechingen, Germany) in 10.5%, VBX (W. L. Gore & Associates, Flagstaff, Arizona) in 5.6%, and iCover (iVascular, Barcelona, Spain) in 1.4%. A second bridging stent (Viabahn; W. L. Gore & Associates, Flagstaff, Arizona) was used in 14 implants. Technical success was achieved in 71/74 procedures (96.0%); three failures occurred because the IIA could not be catheterized. In successful cases, the landing zone was the main trunk of the IIA in 58 and the posterior branch in 13. Concomitant fenestrated or branched aortic repair was performed in 17 procedures (23%). Percutaneous femoral access was used in 87.8%, and transbrachial access was selectively used in 23% of procedures to facilitate iliac branch cannulation and bridging stent deployment, while all aortic endografts were implanted via femoral access. The median contrast volume was 180 mL (110–250), the fluoroscopy time was 35 min (25–50), and the radiation dose–area product was 21,489 Gy·cm^2^ (18,543–30,093). Procedural parameters and anesthesia types are summarized in Table 4. Procedures involving concomitant suprarenal repair were associated with numerically longer procedural duration, higher contrast volume, and increased radiation exposure compared with isolated iliac branch implantation or standard EVAR with iliac branch device; however, none of these differences reached statistical significance. The median procedural time was 160 (105–200) min in patients without concomitant aortic repair versus 120 (110–155) min in those undergoing concomitant aortic repair (*p* = 0.37). The median contrast volume was 180 (110–250) mL versus 150 (110–240) mL (*p* = 0.74), and the median dose–area product was 21,408 (18,692–30,100) Gy·cm^2^ versus 22,400 (18,340–22,540) Gy·cm^2^ (*p* = 0.57), respectively. One patient (1.4%) met KDIGO criteria for acute kidney injury.

The median follow-up was 19 months (6–36). The follow-up duration differed between groups, with a median of 24 (8–36) months in patients without concomitant aortic repair and 12 (3–18) months in those undergoing concomitant aortic repair (*p* = 0.013). All patients attended the 1-month follow-up; 66 patients attended the 6-month follow-up visit. A total of 56 patients attended the one-year follow-up visit while 46, 29, and 8 attended the two-, three-, and four-year follow-up visit, respectively.

Four branch instability events occurred (5.4%): three occlusion/stenoses and one bridging-stent detachment. All were managed endovascularly with additional relining. Seven endoleaks (9.5%) were detected: one type Ib, two type II, two type IIIa, and two type IIIc; one type Ib and one type IIIa endoleak required reintervention. The overall reintervention rate was 6.8%; five procedures were needed: two relinings for type III endoleak, one extension into the external iliac artery for type Ib endoleak, one treatment of a type II endoleak, and one femoro-femoral crossover bypass for bridging-stent detachment. Eighteen patients (24.3%) experienced aneurysm sac regression > 5 mm. Five patients (6.8%) reported transient gluteal claudication that resolved within 3 months. No cases of acute respiratory failure were observed. Only one patient developed acute kidney injury, without need for dialysis. Detailed outcomes are listed in Table 5. Fourteen patients (20%) presented with an internal iliac artery aneurysm. The presence of an IIA aneurysm influenced distal landing zone selection, with posterior branch sealing being more frequently adopted in this subgroup (9/14, 64%) compared with patients without IIA aneurysm (4/60, 7%). Despite this difference in landing strategy, bridging stent length did not differ between patients with and without IIA aneurysm (median 57 (57–57) mm in both groups; *p* = 0.56). Importantly, the presence of an IIA aneurysm was not associated with increased branch instability (7.1% vs. 5.0%; *p* = 0.58) nor with a higher incidence of endoleak (14.3% vs. 8.3%; *p* = 0.61).

Seven patients died during follow-up (10%); none died within 30 days. Causes were COVID-19 pneumonia (*n* = 1), acute coronary syndrome (*n* = 2), melanoma (*n* = 1), gastric cancer (*n* = 1), stroke (*n* = 1), and unknown (*n* = 1). Bridging stent detachment occurred after initial technical success and was therefore considered a post-operative complication rather than a procedural failure. There were no aneurysm-related deaths.

**Figure 1:**
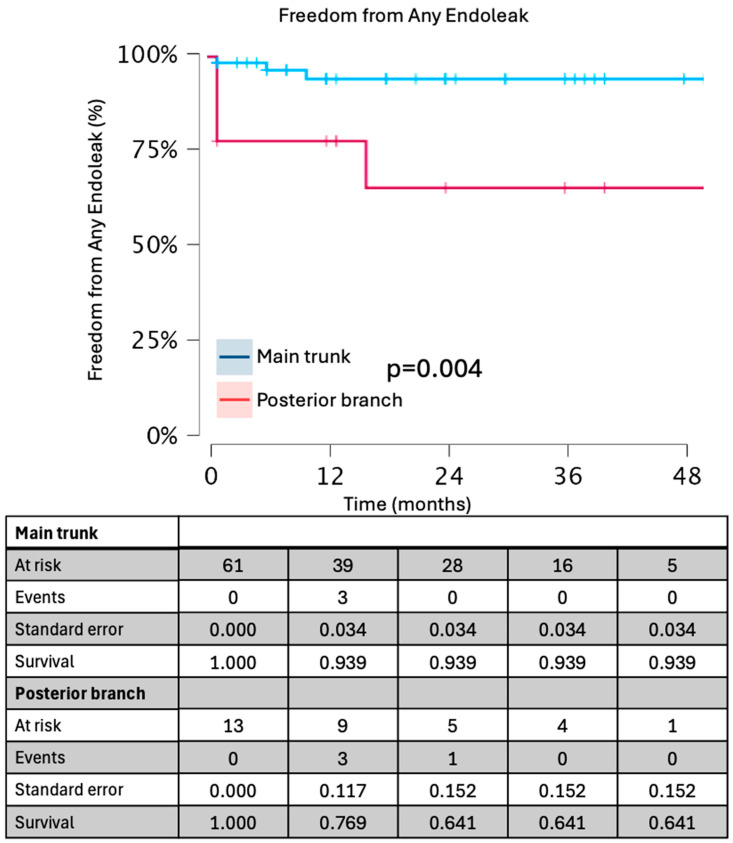
Freedom from any endoleak in groups based on landing zone.

**Figure 2:**
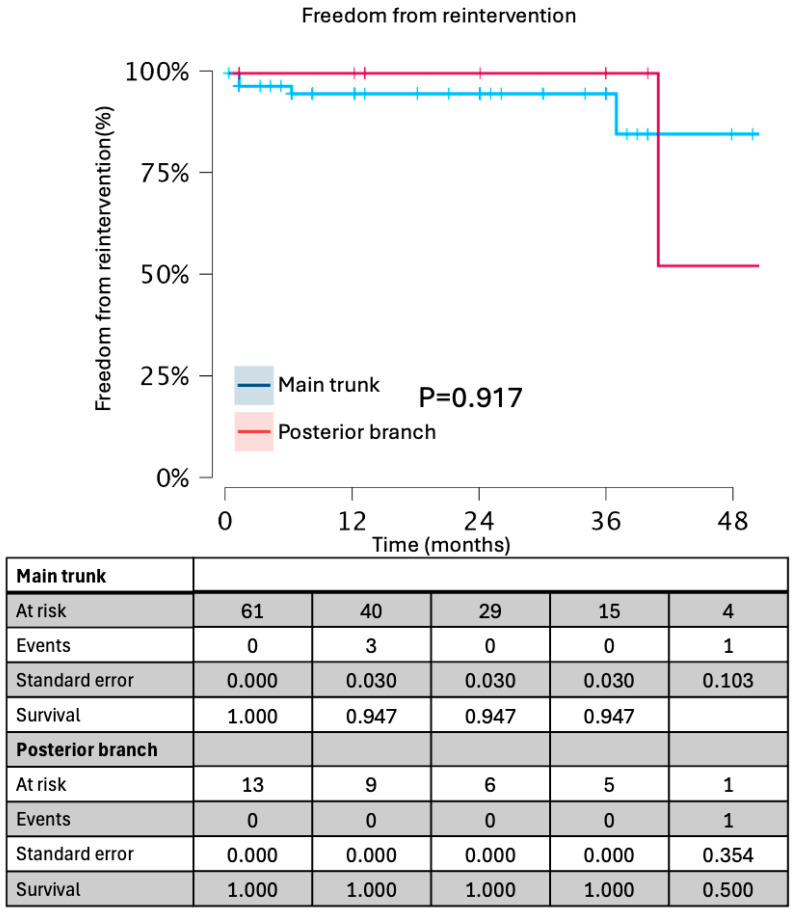
Freedom from reintervention in groups based on landing zone.

Kaplan–Meier curves for freedom from branch instability, any endoleak, and reintervention are presented in Figure 1 and Figure 2.

Survival estimates were truncated at 48 months, beyond which fewer than 10 subjects remained at risk. At 48 months, freedom from branch instability was 92% (95% CI 77–100%) for main-trunk landings and 89% (60–100%) for posterior-branch landings (log-rank *p* = 0.69). Freedom from any endoleak at 48 months was 87% (75–95%), and freedom from reintervention was 93% (83–98%). Rates of branch instability, any endoleak, device-related reintervention, and gluteal claudication were comparable between patients undergoing concomitant aortic repair and those treated with isolated iliac branch implantation or standard EVAR with an iliac branch device.

Log-rank test stratified by concomitant suprarenal aortic repair did not demonstrate significant differences in freedom from branch instability, endoleak, or reintervention between groups (all log-rank *p* > 0.05).

## 4. Discussion

This multicenter series represents, to our knowledge, one of the largest datasets examining mid-term performance of the E-Liac iliac branch device offering mid-term data from a heterogeneous population. EVAR has become the preferred strategy for abdominal and aorto-iliac aneurysms in current European guidelines [16]; however, extension of aneurysmal disease into the iliac bifurcation is frequent, and in many patients, a suitable sealing zone is only available in the external iliac artery. In this setting, intentional coverage or embolization of the internal iliac artery (IIA) has historically been used to secure distal fixation but is associated with substantial pelvic ischemic morbidity [7]. Preservation of hypogastric flow has also long been advocated for in open aorto-iliac repair, where reimplantation or bypass of the internal iliac artery has been used to reduce pelvic ischemia. IBDs translate this established surgical principle into a less invasive endovascular solution. These data provide a robust reason for the current recommendation to preserve at least one hypogastric artery whenever feasible. Subsequent series reported that hypogastric revascularization with side-branch endografts reduced pelvic ischemia compared with hypogastric exclusion, while maintaining comparable early safety [17].

Within this landscape, the E-Liac stent-graft represents a newer-generation IBD characterized by a self-expandable asymmetric design intended to improve trackability and adapt to challenging iliac anatomies. Early single-center experiences by Anton et al. and Dueppers et al. (ABRAHAM study) reported technical success rates above 90% and acceptable early patency when E-Liac was used either for aorto-iliac aneurysms or for exclusion of hypogastric aneurysms, although the cohorts were small and follow-up limited to 12–24 months [18]. More recently, the PLIANT II registry has provided real-world, multicenter evidence in 295 patients, documenting technical success of 93.1%, 12-month clinical success of 91.2%, and a Kaplan–Meier survival of 96.7% with high freedom from type I/III endoleak and IIA/EIA occlusion [19].

Unlike other commercially available iliac branch systems—which require coupling to a bifurcated abdominal endograft—the E-Liac is currently the only IBD with a regulatory indication for isolated use in common iliac artery (CIA) aneurysms, without mandatory simultaneous deployment of aortic bifurcated EVAR. This feature is clinically relevant for several reasons; it reduces aortic coverage, makes the procedure faster, and has potential economic implications, such as the avoidance of a bifurcated aortic endograft, substantially reducing implant cost without compromising the preservation of hypogastric perfusion. Two patients underwent stand-alone E-Liac implantation for isolated common iliac artery aneurysms, with technical success and without early device-related complications. However, the small number of cases precludes any inference regarding comparative outcomes. The indication for isolated iliac branch implantation remains narrow, and larger dedicated series would be required to assess durability and clinical benefit.

With this background, the present CAMPARI study adds multicenter real-world data from five high-volume vascular units, focusing on 74 E-Liac devices implanted in 69 patients with complex aorto-iliac or isolated iliac aneurysms [20]. Notably, all our instability events were successfully managed endovascularly, and no aneurysm-related deaths occurred, further corroborating the safety profile of the E-Liac platform. Although pre-existing contralateral internal iliac artery occlusion was not systematically recorded, the absence of severe pelvic ischemic complications supports effective hypogastric preservation in the present cohort.

Our rate of pelvic ischemic symptoms in our cohort (6.8% transient buttock claudication) is clinically relevant when compared with the historical burden of symptoms following hypogastric exclusion. These results should be interpreted in the context of the existing literature, where lower rates have been reported in more selected cohorts or after unilateral hypogastric exclusion. Importantly, all cases in the present series were transient and resolved without reintervention [21].

One distinctive aspect of CAMPARI is the analysis of distal landing in the main IIA trunk versus the posterior branch, although interpretation of outcomes according to distal landing zone should be viewed in light of the limited number of posterior-branch implantations in this cohort (*n* = 13). The large international R3OYAL registry, encompassing 221 IBD branches, reported no difference in early technical success or composite branch instability between anterior and posterior divisional landings but significantly higher primary and secondary patency at three years when the distal seal was in the posterior division (primary patency 96% vs. 81%; secondary 97% vs. 81%) [22].

In our E-Liac-only cohort, freedom from branch instability at four years was comparable between main-trunk and posterior-branch groups, with a nonsignificant trend towards fewer occlusions in posterior landings; these data, however, should be interpreted cautiously because a limited subset of patients had posterior landing in our cohort, making the estimates unstable. Although our sample is underpowered to detect moderate differences, the concordant direction of effect with R3OYAL suggests that the posterior division is at least not disadvantaged and may represent a preferred landing option when the main trunk is short, aneurysmal, or diseased. From a practical standpoint, these data support a more liberal use of posterior-branch landing with E-Liac in anatomies where a main-trunk seal would otherwise be borderline.

Bridging stent selection is another critical determinant of branch durability. In our study, E-Ventus balloon-expandable stents were used in >75% of primary bridging positions, with Viabahn self-expandable stents reserved for secondary extensions. This mirrors the practice reported by Bracale et al., who demonstrated high early patency and low reintervention rates for E-Ventus as a bridging stent in IBD and F/BEVAR procedures, with patency ~97% at mid-term follow-up [23].

The overall endoleak rate of 9.5% (with 2 type III and 1 type Ib events requiring reintervention) and reintervention rate of 6.8% compare well with IBD series using other platforms, where reinterventions of 8–15% at mid-term are common. Our 6.8% reintervention rate at a median of 19 months is reassuring and suggests that, when used according to IFU, the E-Liac device can deliver at least equivalent, and possibly superior, durability compared with other IBD systems. A dedicated analysis of patients with internal iliac artery aneurysms provides additional insight into device behavior in more complex iliac anatomy. In this subgroup, posterior branch landing was more frequently required, reflecting limited availability of a healthy main trunk for distal sealing. Nevertheless, neither bridging stent length nor branch-related outcomes differed from those observed in patients without IIA aneurysm. IBD-related outcomes were not adversely affected by the presence of concomitant aortic repair, despite the higher procedural complexity associated with fenestrated or branched EVAR. Although these patients required longer and more technically demanding procedures, rates of branch instability, endoleak, and reintervention remained comparable to those observed in isolated iliac or standard EVAR cases.

### Study Limitations

CAMPARI’s retrospective design is subject to inherent biases, although consecutive enrolment and prospective data collection in each center mitigate selection bias. The number of patients in whom hypogastric preservation was considered but abandoned due to unfavorable anatomy was not systematically captured, representing an inherent limitation of this retrospective analysis. Baseline sexual function was not systematically assessed, which limits the interpretation of sexual outcomes. Follow-up, while longer than in many E-Liac reports, remains insufficient to fully characterize very late failures, especially in high-risk patients with progressive iliac degeneration. Moreover, the absence of a contemporaneous comparator cohort treated with alternative IBD platforms or with non-branch techniques (bell-bottom, sandwich/chimney configurations) precludes direct comparative effectiveness conclusions. Finally, the relatively small posterior-branch subgroup limits statistical power to robustly confirm the apparent equivalence of landing zones.

Despite these limitations, CAMPARI complements and extends the current evidence base. Together with PLIANT II and emerging long-term single-center cohorts [23], it supports E-Liac as a safe and durable option for the endovascular treatment of aorto-iliac and isolated iliac aneurysms, with low pelvic ischemic morbidity, high branch patency and a modest reintervention burden. These results advocate for broader adoption of E-Liac in anatomically suitable patients and support its inclusion as a benchmark device in future comparative and cost-effectiveness studies.

## 5. Conclusions

In this multicenter series, the E-Liac demonstrated high technical success, durable branch patency, and low reintervention rates, with excellent preservation of pelvic perfusion and no aneurysm-related mortality. The E-Liac is currently the only iliac branch device approved for stand-alone implantation in isolated common iliac artery aneurysms; however, in the present series, this approach was applied in only two patients and is therefore reported as a technical consideration rather than an outcome-related finding. Transient buttock claudication occurred in a small proportion of patients, with no persistent pelvic ischemic complications. Although these findings support the E-Liac as a robust and adaptable IBD platform, longer-term studies are required to confirm durability beyond mid-term follow-up.

## Figures and Tables

**Table 1:** Baseline clinical features (*n* = 69 patients). eGFR = estimated glomerular filtration rate.

Characteristic	Value
Age (years)	72.5 (67.2–78.8)
Male, *n* (%)	68 (98.6)
Female, *n* (%)	1 (1.4)
Body mass index (kg/m^2^)	26.0 (24.8–27.6)
Current/former smoker, *n* (%)	50 (72.5)
Hypertension, *n* (%)	62 (89.9)
Dyslipidemia, *n* (%)	49 (71.0)
Coronary artery disease, *n* (%)	25 (36.2)
History of myocardial infarction, *n* (%)	13 (18.9)
Chronic obstructive pulmonary disease, *n* (%)	25 (36.2)
Peripheral artery disease, *n* (%)	6 (8.7)
Chronic kidney disease, *n* (%)	7 (10.1)
Diabetes mellitus, *n* (%)	14 (20.3)
History of stroke, *n* (%)	0 (0.0)
Prior aortic repair, *n* (%)	16 (23.2)
Baseline eGFR (mL/min/1.73 m^2^), mean ± SD	72.5 ± 18.9
ASA class, *n* (%)	II: 4 (5.7); III: 47 (67.1); IV: 19 (27.1)
SVS score, mean ± SD	4.27 ± 2.50

**Table 2:** Baseline anatomical features (*n* = 69 patients).

Measurement	Value
Infrarenal aortic diameter (mm)	44.0 (31.9–54.8)
CIA maximum diameter (mm)	34.0 (30.0–40.8)
CIA minimum diameter (mm)	14.3 (12.0–17.8)
CIA length (mm)	57.0 (51.0–68.0)
IIA maximum diameter (mm)	9.8 (8.0–12.0)
IIA minimum diameter (mm)	7.4 (6.8–8.0)
IIA length (mm)	31.5 (22.2–39.8)
Aorta low renal to bifurcation (mm)	120.5 (108.2–153.8)
Aortic bifurcation diameter (mm)	25.8 (22.0–33.6)

**Table 3:** Bridging stent graft details (*n* = 74 devices).

Parameter	Value
Primary stent: E-Ventus, *n* (%)	57 (77.0)
Primary stent: BeGraft, *n* (%)	9 (12.2)
Primary stent: VBX, *n* (%)	5 (6.8)
Primary stent: iCover, *n* (%)	1 (1.4)
Primary stent: unspecified, *n* (%)	2 (2.7)
Secondary stent (Viabahn) used, *n* (%)	14 (18.9)
No secondary stent, *n* (%)	60 (81.1)
Bridging stent diameter (mm)	9.0 (8.0–9.0)
Second stent diameter (mm)	9.0 (8.0–9.0)
Bridging stent length (mm)	57.0 (57.0–57.0)
Second stent length (mm)	57.0 (57.0–57.0)
Total stent length (mm)	57.0 (57.0–57.0)

**Table 4:** Peri-operative parameters (*n* = 74 devices). Gy = gray.

Parameter	Value
Concomitant fenestrated/branched aortic repair, *n* (%)	17 (23%)
Technical success, *n*/N (%)	71/74 (96%)
Distal landing zone: main trunk, *n* (%)	61 (82%)
Distal landing zone: posterior branch, *n* (%)	13 (18%)
Brachial access, *n* (%)	17 (23%)
Percutaneous femoral access, *n* (%)	65 (88%)
General anesthesia, *n* (%)	14 (19%)
Spinal anesthesia, *n* (%)	51 (69%)
Local anesthesia, *n* (%)	9 (12%)
Contrast volume (mL)	180 (110–250)
Procedural time (min)	150 (110–198)
Fluoroscopy time (min)	35 (25–50)
Dose–area product (Gy·cm^2^)	21,489 (18,543–30,092)
Radiation air kerma (mGy)	810 (608–945)
Hospital stay (days)	4 (4–6)

**Table 5:** Outcomes (*n* = 74 devices).

Outcome	Value
Median follow-up (months)	19.5 (6.0–36.0)
Branch instability, *n* (%)	4 (5.4%)
Any endoleak, *n* (%)	7 (9.5%)
Type I endoleak, *n* (%)	1 (1.4%)
Type II endoleak, *n* (%)	4 (5.4%)
Type III endoleak, *n* (%)	2 (2.7%)
Reinterventions, *n* (%)	5 (6.8%)
Gluteal claudication, *n* (%)	5 (6.8%)
Acute kidney injury, *n* (%)	1 (1.4%)
Acute respiratory failure, *n* (%)	0 (0%)
Sac regression > 5 mm, *n* (%)	18 (24.3%)
All-cause mortality, *n* (%)	7 (9.5%)
30-day mortality, *n* (%)	0 (0%)

## Data Availability

The raw data supporting the conclusions of this article will be made available by the authors on request.
